# Advances in Sensors Applied to Agriculture and Forestry

**DOI:** 10.3390/s110908930

**Published:** 2011-09-15

**Authors:** Gonzalo Pajares

**Affiliations:** Department of Software Engineering and Artificial Intelligence, Facultad de Informática, Universidad Complutense, 28040 Madrid, Spain; E-Mail: pajares@fdi.ucm.es; Tel.: +34- 913947546; Fax: +34 913947547

In agriculture and forestry, the need to increase production and the simultaneous efforts to minimize the environmental impact of agricultural production processes and save costs find in sensor systems the best allied tool. The use of sensors helps exploit all available resources appropriately and to apply hazardous products moderately. When nutrients in the soil, humidity, solar radiation, density of weeds and a broad set of factors and data affecting the production are known, this situation improves and the use of chemical products such as fertilizers, herbicides and other pollutants can be reduced considerably. Part of this knowledge allows also monitoring photosynthetic parameters of high relevance for photosynthesis. Most of the associated activities fall within the scope of what it is called Precision Agriculture, an emerging area receiving special attention in recent years.

In forest management, a branch of forestry, a number of activities are oriented towards wood production or forest inventories with the aims of controlling parameters of interest such as diameter of trees, height, crown height, bark thickness, canopy, humidity, illumination, CO_2_ transformation among others, always with the goal of environmental sustainability with high social impact. Sensors offer solutions for controlling and monitoring production in forest trees while the same time costs are minimized by applying selective treatments thanks to the boom of these technologies. Additionally, during the post-production process, including transportation, storage, packing, selection, classification or distribution, among others, the use of sensors is of vital importance for minimizing costs and negative environmental impact, allowing energy savings or minimizing the application of chemical products.

Farmers, researchers and technical manufacturers are joining important efforts. always trying to find more efficient solutions for solving different problems or for improving current production or processes. The exponential growth of sensors capabilities and technologies allow exploiting these capabilities and the above goal becomes feasible. The works published on this special issue are a clear demonstration of these assertions. In what follows the relevant sensor-based technologies and applications proposed on this special issue are presented. *Image-based sensors* are a powerful tool for different purposes, including climate variability and temporal analysis of crop field areas, providing added value for crop production. This kind of sensors can also be used for analysis and quantification of crop damage. Analysis of soil coverage by residues is possible and desirable for controlling subsequent fertilizations. Crop and weed segmentation techniques based on images are of special interest in Precision Agriculture. The combination of laser and image cameras allows controlling the quality of apples under storage. Image sensors are also used for navigation in orange groves. Three dimensional modeling of tea-shoots based on images is also possible. The use of hyper-spectral and chlorophyll fluorescence imaging allow analyzing the integrity of infected wheat ears. The use of stereoscopic images captured with fish-eye lenses is useful for forest inventories. Multi-temporal is feasible in sugarcane by observing TerraSAR-X images. Image-based sensing technologies and photoconductive cells are conveniently combined for studying buoyant fluorescent microspheres as particle tracers in turbid water flows from the point of view of agricultural applications.

*Sensor networks* allow collecting different types of *in-situ* information which can be conveniently exploited for controlling crop production or monitoring ecosystems by analyzing different variables, such as light, temperature, humidity or climatological and anthropological events, among others. This information can be acquired by sensors deployed in different countries or areas and processed remotely, including web technologies. Sensors networks combined with image-based devices allow monitoring pests and diseases in vineyards. Analysis of biomass quality is also possible with sensor networks. Specific and dedicated sensor networks platforms have been applied in Precision Agriculture.

*Specific sensors* for soil moisture in both, agricultural and forest environments are also of special interest for analysis of plants.

Unmanned Aerial Vehicles (UAV), equipped with different sensors, in collaboration with ground-based sensors have become a powerful tool for early fire forest detection and posterior monitoring. This combination of sensors has also been applied for crop monitoring under a wireless sensor network architecture.

*LIDAR sensors* are used to obtain dynamic measurements to estimate fruit-tree leaf area and combined with GPS have been applied for 3D map generation in vine plantations. *Laser and hyperspectral* data are used for tree classification, including coniferous and deciduous trees. 3-D modeling of tomato canopies is obtained through high-resolution portable scanning LIDAR. Airborne LIDAR data are processed for estimating biomass in alpine forests.

*Agricultural robotics systems* are continuously expanding and the design of efficient sensor architectures for task classification, communication and control results of great relevance. The communication can be established among different vehicles or between different sensors mounted on a unique ground or aerial mobile unit, where sensors must be conveniently integrated.

An interesting area is the one related to *autonomous or remotely guided agricultural tractors*. For positioning a tractor in the field only GPS can be used as the unique sensor if the receiver is placed ahead of the tractor. Sensors based on augmented reality technology are useful from the point of view of autonomy. Guidance of a tractor by means of an electromiographic-based human-machine interface is vital for disabled people.

*Raman* and *Fourier Transform Infrared spectroscopy* has been used for assessment of structural differences of celluloses of various origins.

*Soft water level-based sensors* for characterizing the hydrological behavior in agriculture catchments have been used with promising prospects.

*Capacitance probes* are suitable for measurement of soil moisture in tropical areas.

The use of *methodologies* for boom sprayer regulation has been successfully applied; this was intended to guarantee that the dose of the product applied per surface unit is similar across the field.

*Reflectometers* can determine moisture content in oil palm fruits.

*Ultrasonic ranging sensors* are used for analyzing apple tree canopies.

*Optoelectronic sensors* for weed detection in wide row crops have been analyzed in terms of accuracy and feasibility.

*pH soil-based sensors* allow measurements of variables in the soil oriented toward crop productivity.

*Eddy covariance sensors* are applied to quantify carbon metabolism of peatlands and also regional and global analysis of observations from micrometeorological tower sites.

A solar energy powered *autonomous wireless actuator* has been designed for irrigation systems in agriculture.

*Fluorescence-based optical sensor* for plant constituent assessment was used to monitor grape maturation by specifically monitoring anthocyanin accumulation.

The measurement of the leaf temperature of forests or agricultural plants is an important technique for the monitoring of the physiological state of crops. The *infrared thermocouple* is a convenient device due to its fast response and nondestructive measurement technique.

Classification of seeds through an *acoustic* sensor is possible analyzing sound absorption spectra.

An *FPGA-based* fused smart sensor for real-time plant transpiration monitoring is possible.

Automated point *dendrometers* to analyze tropical treeline stem growth has been applied in forest inventories.

*Optical* and *microwave sensors* are suitable for characterizing olive grove canopies.

